# Habits: bridging the gap between personhood and personal identity

**DOI:** 10.3389/fnhum.2014.00330

**Published:** 2014-05-21

**Authors:** Nils-Frederic Wagner, Georg Northoff

**Affiliations:** ^1^Royal Ottawa Health Care Group, Mind, Brain Imaging and Neuroethics, Institute of Mental Health Research, University of OttawaOttawa, ON, Canada; ^2^Taipei Medical University-Shuang Ho Hospital, Brain and Consciousness Research CenterNew Taipei City, Taiwan

**Keywords:** habits, personhood, personal identity, decision-making, default-mode network, resting state, fMRI

## Abstract

In philosophy, the criteria for personhood (PH) at a specific point in time (synchronic), and the necessary and sufficient conditions of personal identity (PI) over time (diachronic) are traditionally separated. Hence, the transition between both timescales of a person's life remains largely unclear. Personal habits reflect a decision-making (DM) process that binds together synchronic and diachronic timescales. Despite the fact that the actualization of habits takes place synchronically, they presuppose, for the possibility of their generation, time in a diachronic sense. The acquisition of habits therefore rests upon PI over time; that is, the temporal extension of personal decisions is the necessary condition for the possible development of habits. Conceptually, habits can thus be seen as a bridge between synchronic and diachronic timescales of a person's life. In order to investigate the empirical mediation of this temporal linkage, we draw upon the neuronal mechanisms underlying DM; in particular on the distinction between internally and externally guided DM. Externally guided DM relies on external criteria at a specific point in time (synchronic); on a neural level, this has been associated with lateral frontal and parietal brain regions. In contrast, internally guided DM is based on the person's own preferences that involve a more longitudinal and thus diachronic timescale, which has been associated with the brain's intrinsic activity. Habits can be considered to reflect a balance between internally and externally guided DM, which implicates a particular temporal balance between diachronic and synchronic elements, thus linking two different timescales. Based on such evidence, we suggest a habit-based neurophilosophical approach of PH and PI by focusing on the empirically-based linkage between the synchronic and diachronic elements of habits. By doing so, we propose to link together what philosophically has been described and analyzed separately as PH and PI.

## Introduction

What is a person? More precisely, which conditions are necessary for an entity to be a person at a discrete point in time; or, which features define an entity synchronically as a person? It is important to shed light on the constitutive features of personhood in order to be able to determine how persons persist, since entities of different kinds persists in different ways. Once the constitutive features of personhood have been settled, one can ask what it takes for the *same* person to exist at different times. Since John Locke added a chapter on identity and diversity to the second edition of his “Essay Concerning Human Understanding” (Locke, 1694/1975), these questions have been intensely discussed in philosophy, as well as in related disciplines.

In the philosophical discussion, traditionally, there has been a separation between the criteria of personhood and the necessary and sufficient conditions of personal identity. That is, the *synchronic* and the *diachronic* dimension of a person's life have mostly been discussed and analyzed separately. The traditional view in philosophy of mind is that the constitutive conditions of personhood at a specific point in time and the criteria for persons to persist through time are neither identical nor coextensive. What makes someone a person at time t_1_ does not account for what makes this person persist; however, quite frankly, these two dimensions of a person's life are closely related. Only if we know the conditions of personhood, can we give a compelling account of personal identity over time. Similarly, only if we have an idea of how persons persist, can we coherently analyze their synchronic dimension. This is so, as we will elaborate throughout this paper in more detail, because at least one constitutive feature of personhood—namely self-reflectiveness, particularly in its role of planning agency—involves a temporal dimension. Disregarding the temporal transition from personhood to personal identity leaves not only a gap in an encompassing theory of what constitutes a person's life as a whole, but also limits the explanatory scope of each dimension on its own. It is for this reason that theories of personal identity must at least implicitly presuppose a view of personhood; and accounts of personhood must at least implicitly consider how personal identity is constituted. Our attempt is to offer some empirically informed suggestions of how this implicit linkage between personhood and personal identity can be elucidated. We believe that personal habits serve an explanatory purpose in how these different temporal dimensions of a person's life are linked. Yet, our hypothesis does not come out of the blue. In the philosophy of action there have been some attempts to address this issue. Particularly, Frankfurt (1982, 1988), Korsgaard (1996, 2009), and Bratman (2000) offer conceptual resources of how human agency involves reflection and planning, which implies both the synchronic and diachronic dimension of a person's life. In the discussion section, we draw on some of Bratman's conceptual work and approximate how our hypothesis is in line with his account, and further, how it can be fruitfully complemented with the empirical evidence we discuss.

To start with, we will give a brief overview of the paradigmatic approaches in philosophy of both the synchronic question of personhood and the diachronic question of personal identity. For that purpose, quite a bit of conceptual ground-clearing will be necessary. We will reconstruct the criteria for personhood and personal identity that have been claimed to be most plausible. This discussion will suggest that the separate analysis of personhood and personal identity leaves an unnecessary gap between the synchronic and the diachronic dimension of a person's life. Subsequently, in order to make an attempt to bridge this gap, we will shed light on the conceptual role that personal habits play in the linkage between personhood and personal identity. In light of this conceptual analysis, we further investigate how the temporal linkage between synchronic and diachronic aspects of a person's life is mediated empirically. Finally, we will outline an account that shows how this empirical mediation can bridge the gap between personhood and personal identity. In so doing, we will analyze the synchronic dimension of personhood and the diachronic dimension of personal identity in the realm of decision-making, which will show how habits can be considered to reflect a balance between internally and externally guided decision-making. More specifically, we will show how decision-making in form of habitual behavior already implicates a particular balance between the diachronic and synchronic aspects of a person's life, thereby linking together these two different temporal dimensions.

## Personhood and its synchronic characterization

What do persons have that non-persons don't have? The philosophical goal has largely been to identify a set of mental features possessed by all and only persons. These features, both traditionally and in recent philosophical discussions, are determined first and foremost by higher-order cognitive functions. It is fairly agreed upon the view that a person is someone who acts from reasons. This conception of personhood has a long tradition, reaching back to John Locke who famously regarded the concept of a person as a “forensic term.” Locke says, a person is “a thinking intelligent being, that has reason and reflection, and can consider itself as itself, the same thinking thing, in different times and places” (Locke, 1694/1975, p. 335). Locke established this rationality-based understanding of personhood as a foundation for his account of personal identity over time. This view has a great number of modern day successors, sometimes referred to as “Neo-Lockeans” (Shoemaker, 1970, 1984, 1997, 1999; Parfit, 1971, 1984, 2007; Perry, 1972; Lewis, 1976; Nozick, 1981; Nagel, 1986; Noonan, 2003).

With regard to the moral consideration of human life, Immanuel Kant makes similar remarks when he states that “every rational being exists as an end in himself and not merely as a means to be arbitrarily used by this or that will … rational beings are called persons inasmuch as their nature already marks them out as ends in themselves” (Kant, 1785/2012, p. 428). In the “Lectures on Anthropology,” Kant once again emphasizes that moral considerations are closely related to rationality, he states: “The fact that the human being can have the representation “I” raises him infinitely above all the other beings on earth. By this he is a person. … [T]hat is, a being altogether different in rank and dignity from things, such as irrational animals, with which one may deal and dispose at one's discretion” (Kant, 1798/2012, p. 127). Rationality, in Kant's eyes, is the foundation for human dignity which distinguishes us from animals and holds us responsible for our actions. In the contemporary debate, Christine Korsgaard puts this point forward, combining elements of Kant, Plato and Aristotle (Korsgaard, 2009). Peter Singer is another prominent advocate of a rationality-based view of personhood. Singer sees the special moral value in a person's life preserved in four features: (1) Being rational and self-consciously aware of oneself as an extended body existing over an extended period of time. (2) Having desires and making plans. (3) Containing a necessary condition for the right to life that one desires to continue living. (4) Being autonomous (cf. Singer, 1979, pp. 78–84).

In what follows, we focus on the prevailing claim that rationality is the conceptual starting point for personhood. This has been fleshed out paradigmatically by Daniel Dennett, who aims to define necessary conditions of personhood that are fundamentally based on our cognitive abilities. In his seminal paper, Dennett claims that

“being rational is being intentional is being the object of a certain stance. These three together are necessary but not sufficient conditions for exhibiting the form of reciprocity that is in turn a necessary but not sufficient condition of having the capacity for verbal communication, which is the necessary condition for having a special sort of consciousness, which is …, a necessary condition of moral personhood (Dennett, 1976, p. 179).”

Rationality is established as the necessary condition to acquire the additional features that together make up personhood. Therefore, all other features of personhood in Dennett's account can be seen as derivative to rationality. Dennett explicitly calls rationality “the *first* and most obvious theme” (Dennett, 1976, p. 177) of personhood. Subsequently, Dennett gives six defining conditions of personhood—he calls them *themes*. They can be summed up in the particular order of their appearance as listed in Table 1.

**Table 1:** **Synchronic criteria of personhood**.

Rationality Conscious mental states and intentionality Being the subject of a special stance or attitude of regard by other persons Being able to give that regard back to others (reciprocity) Capacity for verbal communication Self-consciousness

Dennett aims to account for the rationality-based conditions that need to be fulfilled in order to ensure that an entity at a given point in time qualifies as a person. This account is synchronic because it is not concerned with the criteria that are necessary and sufficient for a person to persist through time. To illustrate the claim that Dennett's account is synchronic rather than diachronic, consider the following example. An entity X at time t_1_ is a person by virtue of him meeting the criteria stated in Table 1. At time t_2_ X continues to be a person because he still meets the criteria in Table 1. However, X at time t_2_ might have lost all the memories, intentions, preferences, desires and so forth that he possessed at time t_1_, and is therefore no longer the *same* person; nevertheless X is still *a* person. In other words, the criteria for someone to be a person at a given time and the criteria for a given person to persist through time are different.

Dennett's aim is to show how the features stated in Table 1 are necessary conditions of personhood, dependent on each other. Rationality is seen as the starting point for the ascription of conscious mental states to other persons and intentionality. By claiming that persons are attributed to having states of consciousness, Dennett includes that persons have “Intentional predicates” (Dennett, 1976, p. 177). That is to say, in order to think or act intentionally, a person has to decide to treat the entity whose behavior is to be predicted as a rational agent. Subsequently, the person tries to figure out what beliefs that agent ought to have, given its place in the world and its purpose. Then the person figures out what desires it ought to have, and finally the person predicts that this rational agent will act to further its goals in the light of its beliefs. By means of this kind of practical reasoning, the person is able to predict what the rational agent will do (cf. Dennett, 1996, p. 17).

One can easily imagine other intentional systems besides human persons. Dennett gives examples of dogs and chess playing computers. According to Dennett, intentionality is not a sufficient but surely a necessary condition of personhood: “Nothing to which we could not successfully adopt the Intentional stance, with its presupposition of rationality, could count as a person” (Dennett, 1976, p. 180). When Dennett further claims that “whether something counts as a person depends in some way on an attitude taken toward it” (Dennett, 1976, p. 177), he implicitly concedes that personhood is not entirely an intrinsic feature, but to some extend a matter of social ascription. The same holds true for reciprocity, by which Dennett emphasizes that the ascription of personhood is not something that is merely given, but also something that has to be returned. Therefore, reciprocity is the capacity to exhibit higher-order intentions and thus depends on the first three, but not on the fifth and sixth condition (cf. Dennett, 1976, p. 185). To establish verbal communication as a necessary condition of personhood is rather narrow. On these grounds this requirement has been criticized by a great deal of other philosophers. In Dennett's account, verbal communication serves the goal to further link personhood to morality and, by doing so, to exclude non human animals from full personhood. However, this also comes at the cost of excluding, among others, infants. Self-consciousness is another feature that Dennett believes only to be present in humans, and, since it is seen as a precondition for morality, it defines persons as the only beings capable of morality. Self-consciousness depends in Dennett's account on the previous established conditions and, rather surprisingly, not vice versa. In order to substantiate this claim, Dennett adverts to moral responsibility. To be held responsible for an action, Dennett says, a person must have been *aware* of that action: “Because only if I was aware of the action can I say what I was about, and participate from a privileged position in the question-and-answer game of giving reasons for my actions” (Dennett, 1976, p. 191). Once again, the emphasis lies on the rational capacity of acting from reasons and on constituting ourselves by choosing the actions in awareness of our responsibility for them. “The capacities for verbal communication and for awareness of one's actions are thus essential in one who is going to be amenable to argument or persuasion, and such persuasion, such reciprocal adjustment of interest achieved by mutual exploitation of rationality, is a feature of the optimal mode of personal interaction” (Dennett, 1976, p. 191). With reference to Harry Frankfurt's concept of “second-order volitions” (Frankfurt, 1971), i.e., the unique ability of persons to develop volitions *about* other volitions, Dennett points out that reflective self-evaluation is yet another person constitutive feature that is directly dependent (and therefore subsumed under) self-consciousness. Due to our ability of being able to self-reflectively questioning our own beliefs and desires, and eventually agree or refuse them, we move beyond a level of mere informing ourselves about our beliefs and desires toward a deliberative level of an “Anscombian reason-asker and persuader” (Dennett, 1976, p. 193).

Dennett admits that, although all the conditions he has established as being necessary for personhood, one cannot simply assume that their sum is sufficient. This is so, because personhood is an inescapably normative concept and to that extent, when it is applied to categorize entities ontologically, it is a regulative idea (or a heuristic device) rather than an actual achievable goal. However, the reasons Dennett gives for what makes it even in principle very difficult (if not impossible) to find sufficient conditions for personhood are somewhat peculiar. Dennett claims: “There is no objectively satisfiable sufficient condition for any entity's *really* having beliefs, and as we uncover apparent irrationality under an Intentional interpretation of an entity, our grounds for ascribing any beliefs at all wanes, especially when we have (what we always *can* have in principle) a non-Intentional, mechanistic account of the entity” (Dennett, 1976, p. 193 f.). Peculiar about this claim is how fundamental the connection of rationality and the ascription of beliefs are linked in Dennett's account. One could ask why an irrational action, even an action that is averse to a person's apparent beliefs, should make it altogether impossible to still ascribe this belief to the person. Having a belief does not to necessarily entail that a person always acts in accordance with this very belief, unless one assumes that persons are *ipso facto* and above all, rational beings. It seems this is exactly what Dennett intends to claim when he asserts that rationality is the necessary condition for personhood.

Even though philosophers differ in the details concerning the necessary conditions of personhood, rationality is in almost every account fundamental. For the purpose of this paper, we go with this standard view. Albeit, there are alternative approaches in philosophy to what constitutes personhood. Marya Schechtman convincingly argues for a view which is less demanding in terms of cognitive abilities, but rather focuses on the social constitution of personhood as its most salient feature (Schechtman, 2010, 2014).

Having reconstructed the paradigmatic philosophical view of what constitutes persons synchronically, we now turn to ask how persons persist through time.

## Personal identity and its diachronic characterization

If you point to a child on an old photograph of your class, say 20 years ago, and proclaim: “This is me!”—an obvious question pops up: In which way are you related to the child on the photograph that makes it true that you today and the child on the photograph are identical, or the same person over time? This is a question of diachronic personal identity. In order to answer these kinds of questions, we must know the *criterion* of personal identity over time; i.e., the relation between a person at one point in time and a person at another point in time which makes them one and the same person.

When philosophers debate personal identity, they are mostly concerned with *numerical* identity, whereby they mean that, despite of qualitative changes, a person still remains numerically identical, and thus persists through time. For example, a person X radically changed in her personality traits, as well as in her appearance due to a religious conversion. These changes, however, do not make X cease to exist altogether, they rather alter her *qualitative* identity. In questions about numerical identity, we look at two names or descriptions, and ask whether these refer to one and the same person at different times, or rather to different persons. Philosophers focus on numerical identity, since in the concern about our own futures it is this kind of identity that we care about. However much X will change, X shall still be alive, if there will be someone living who will be numerical identical to X. For this reason, some philosophers prefer to use the term *survival* in order to ensure that numerical and not qualitative identity is at issue.

Some concerns have been raised about this understanding of personal identity. Ludwig Wittgenstein famously argued that talking of *identity over time* is, if not false, at least somewhat misleading: “Roughly speaking, to say of *two* things that they are identical is nonsense, and to say of *one* thing that it is identical with itself is to say nothing at all” (Wittgenstein, 1921/1961, p. 5.5303). This understanding applies to numerical identity conditions of basic material entities like stones, but seems too narrow in terms of personal identity. It goes without saying that it is impossible for a single person at two different points in time to be identical to itself in a strict logical sense; especially if taken into account that the human body's cells are constantly replaced. However, this does not seem to be the kind of identity that we are concerned about when we reflect upon personal identity in terms of caring for our own survival. It is closer to what David Wiggins refers to when he talks of the “conditions of persistence and survival through change” (Wiggins, 1967). An understanding of personal identity through change is, both from a pretheoretical point of view and after conceptual analysis, more compelling than to appeal to strict logical identity. For this reason, accounts of personal identity over time allow for persons to change and nonetheless hold on to a broad, i.e., not strict logical, notion of identity.

## Different criteria of personal identity

In the philosophical debate on personal identity, two main opposing strategies evolved in order to account for what is necessary and what is sufficient for a person to persist through time. Therein, personal identity is either based on a *Reductionist* or on a *Non-Reductionist* understanding.

According to reductionist theories, personal identity is reducible to more particular facts about persons and bodies. The approach is to describe a particular relation *R* that accounts for a person X to be identical to a later existing person Y, by virtue of X and Y being *R*-related. In other words: X is one and the same person as Y, if and only if X stands in relation *R* to Y. In principal, Relation *R* is believed to be empirically observable. However, there is major disagreement about what relation *R* consists in. That is to say, philosophers disagree about which particular ingredients determine the relation that constitutes personal identity over time. In the contemporary debate, most philosophers hold one or another form of a reductionist account; typically, either a form of physical/biological reductionism, or more often, a form of psychological reductionism. In what follows, we will discuss the merits and demerits of the most seminal versions of these criteria.

In contrast to reductionist theories of personal identity, non-reductionists believe that personal identity is not reducible to more particular facts about persons and/or bodies, but rather consists in a non-analyzable, or *simple*, further fact. This is why non-reductionist theories are also referred to as “simple views.” Derek Parfit describes the notion of a further fact as “separately existing entities, distinct from our brains and bodies, and our experience” (Parfit, 1984, p. 445). Non-reductionists thus claim that personal identity consists in a special ontological fact, a Cartesian Ego or a soul; or stated in a less antiquated way, the view is that personal identity consists in a mental entity that is neither reducible to neural mechanisms in the human brain, nor to the way in which the human brain relates to its environment and thereby gives rise to consciousness.

In the contemporary discussion in philosophy of mind few philosophers advocate for non-reductionist accounts of personal identity because those accounts are, at least by the majority of philosophers, believed to be metaphysically contentious. It is argued that non-reductionists in the debate on personal identity take an obscure metaphysical belief and inflate it into a conceptual core conviction. We here refer to the term “metaphysical” explicitly in the way in which it is used in current philosophy of mind, and more particular, in the discussion on personal identity. This is not to ignore that metaphysics has very different nuances depending on the philosophical approach, and that it is hardly used in a non contentious way. In the case of personal identity, non-reductionists arguably presuppose a form of substance, or at minimum property dualism. Both these forms of dualism do not find many advocates in the contemporary discussion on personal identity. Substance dualism is a view in philosophy of mind according to which there are two essentially different *substances* in the world: material and immaterial substances. The mind is not just a collection of thoughts, but it is the substance itself that thinks, an immaterial substance over and above its material states. Property dualism is the view according to which there are two essentially different *properties* in the world. Properties—unlike substances—are possessed by someone or something. Property dualists thus hold the view that immaterial properties like mental states are possessed by what is otherwise a purely material thing; for example, a brain.

Granting the aforementioned concerns about non-reductionism, we will not further elaborate on those accounts. Instead, we will focus on the most paradigmatic reductionist accounts of personal identity: the seminal versions of the *psychological* and the *bodily* criterion.

According to the psychological criterion of personal identity, X and Y is one and the same person at different points in time, if and only if, X stands in a *psychological continuity* relation to Y. You are the same person in the future (or past) as you are now if your current beliefs, memories, preferences and so on are linked by a chain of overlapping psychological connections. Among philosophers who advocate for psychological approaches to personal identity there is dispute over several issues: What mental features need to be inherited? What is the cause of psychological continuity, and how do its characteristics have to be? Must it be realized by some kind of brain continuity (cf. Northoff, 2004), or will “any cause” do? The any cause discussion is concerned about the yet counterfactual idea of whether personal identity that is realized by psychological continuity would still hold, even if this continuity would no longer be caused by the brain, but, for example, by a computer program. Another issue is whether a “non-branching clause” is needed, which ensures that psychological continuity holds to only one future person. Why this can become relevant will be explicated in what follows. We will also go over some of the other aforementioned issues hereafter.

Some agreement rests upon a notion of psychological continuity that has been put forward by Derek Parfit and can be seen as a standard account, according to which (Table 2).

**Table 2:** **Diachronic psychological criterion of personal identity**.

We might appeal, either in addition or instead, to various psychological relations between different mental states and events, such as the relations involved in memory, or in the persistence of intentions, desires, and other psychological features. These relations together constitute what I call psychological *connectedness*, which is a matter of degree. Psychological *continuity* consists of overlapping chains of such connections (Parfit, 2007, p. 6).

Mere psychological connectedness does not suffice as a criterion of personal identity because it is subject to the “transitivity objection.” The transitivity requirement of identity states that, if X is identical to Y, and Y is identical to Z, then X must also be identical to Z. Therefore, personal identity cannot consist in mere psychological connectedness. With the appeal to psychological continuity as overlapping chains of psychological connections, the transitivity objections is resolved, since it allows for indirect relations which ensure identity through time. For example, if you as and old man remember what you have done as a middle aged man, but fail to remember what you have done as a young boy, without overlapping chains of psychological connections between the old man and the young boy, you would no longer be identical to the young boy. Since this would violate the transitivity requirement of identity. However, if you as a middle aged man still remember what you have done as a young boy, then, by virtue of overlapping chains of psychological connections, you as an old man are still identical to the young boy, even though you don't have direct access to the young boy's memories anymore. The old man is one and the same person as the young boy because, broadly speaking, they are indirectly linked through the psychological states of the middle aged man. Here it becomes apparent that psychological continuity, particularly in the sense of persisting intentions, desires and other psychological features, not only hold backwards but, as it were, also forwards. When a person envisages herself into the future, she sees herself preserving certain intentions, desires and other psychological features. Only then can she see herself as the same person persisting through time.

According to the bodily criterion of personal identity, X and Y is one and the same person at different points in time, if and only if, X stands in a *bodily continuity* relation to Y. To put it plainly: you are the same person in the future (or past) as you are now (or have been earlier), as long as you continue to have the same body. A slightly modified version of the bodily criterion is *Animalism*; the view according to which you are the same being in the future (or past) as you are now (or have been earlier), as long as you are the same biological organism. Animalists usually deny the significance of personhood for the debate on personal identity. This is one reason animalists invoke in order to distinguish their criterion from bodily continuity criteria.

One might justifiably ask, what—in real life scenarios—is the discrepancy between psychological continuity and bodily continuity views of personal identity? Doesn't psychological continuity coincide with bodily continuity? The different criteria mainly (although not exclusively) start disagreeing in hypothetical cases. Puzzles such as Locke's famous “Prince and the Cobbler,” are still widely discussed in the metaphysical debate on personal identity. Locke asks what would happen if the soul of a prince, carrying with it the consciousness of the prince's past life, were to enter the body of a cobbler. Locke suggests that as soon as the Cobbler deserted by his own soul, everyone would see that he was the same *person* as the prince, accountable only for the prince's actions. But, who would say it was, in Locke's term, the same man, i.e., human animal? With this thought experiment, Locke suggests that persons, unlike human animals, are only contingently connected to bodies. Locke further believes that what constitutes a person, and moreover the *same* person, is consciousness—by which he essentially means the awareness of one's thoughts and actions: “Nothing but consciousness can unite remote existences into the same person” (Locke, 1694/1975, p. 464). Referring to a man he had met who believed his soul had been the soul of Socrates, Locke asks: “If the man truly were Socrates in a previous life, why doesn't he remember any of Socrates' thoughts or actions?” Locke even goes so far as to say that if your little finger is cut off and consciousness should happen to go along with it, leaving the rest of the body, then that little finger would be the person—the same person that was, just before, identified with the whole body (cf. Locke, 1694/1975, pp. 459–460). Therefore, Locke and his modern day successors establish that wherever your mental life goes, that is where you as a person go as well.

Apart from thought experiments, in real life we might consider the case of permanent vegetative state patients to support Locke's thought experiment, inasmuch as it shows that psychological continuity and bodily continuity do not always coincide. Psychological continuity is not necessarily in place whenever a human organism is around. This assertion does not, of course, imply any dualistic assumptions of immaterial sources of psychological continuity; it merely states that not every form of biological continuity of a human organism is sufficient to support psychological continuity. For all we know now, permanent vegetative state patients lack any higher-order mental features that could possibly constitute psychological continuity, albeit, they are biologically alive. Therefore, according to the psychological criterion of personal identity, there is no identity relation between a conscious person that later becomes a vegetative state patient. Advocates of the bodily criterion see things differently. In their view the identity relation still holds because there continues to be bodily continuity between the person that once had a mental life and the human organism that is now in a permanent vegetative state.

Despite all the difficulties within Locke's view, which cannot be discussed, let alone resolved here, the aforementioned puzzle cases, as well as the permanent vegetative state example, support the widely advocated psychological continuity theories of personal identity. Furthermore, our ordinary intuitions in these scenarios support psychological continuity rather than mere bodily/biological continuity as the criterion for personal identity over time.

The different psychological continuity theories, however, share a severe problem. Unlike identity, psychological continuity is not necessarily a one-one relation. For example, fission scenarios, either based on purely hypothetical cases or based on brain bisection (Corpus Callosotomy), as put forward, among others, by Thomas Nagel, show that psychological continuity does not follow the logic of an identity relation (Nagel, 1971). It is possible in principle, and in accordance with empirical evidence, that psychological continuity divides, and thus, that it holds to more than one person. [For an analysis of the empirical plausibility of different accounts of personal identity see Northoff (2001)]. Albeit, as David Lewis and others pointed out, identity is necessarily a one-one relation that can by definition only hold to itself; whereas psychological continuity is only contingently a one-one relation and may become one-many (Lewis, 1976). Therefore, as Bernard Williams took issue with, psychological continuity is unable to meet the metaphysical requirements of an account of personal identity, unless a non-branching clause is added which ensures that psychological continuity is a one-one relation (Williams, 1973). Nevertheless, the addition of such a non-branching clause is not fully convincing either. This is so, because, as Derek Parfit claimed, a non-branching clause has no impact on the intrinsic features of psychological continuity, and is therefore unable to preserve what we believe to be important in identity (Parfit, 1984). An identity relation can by definition apply to only one person. This leads Parfit to the conclusion that in the end, personal identity is neither here nor there, or as he famously puts it: “Identity is not what matters” after all because the importance we ascribe to it is merely contingent. It seems to be entirely dependent on psychological continuity, which, as mentioned before, is logically not an identity relation. When we are concerned with our survival, what we really should care about is, in Parfit's view, psychological continuity, whether or not it coincides with identity. [For a suggestion of how this problem can be tackled in terms of personal identity in practical reality see Wagner (2013). For a thoughtful critical discussion of Parfit's criterion see Teichert (2000)].

Hereafter, we will put forward the hypothesis that habits can serve to bridge the gap between synchronic and diachronic aspects of a person's life. In order to give a prospect of this hypothesis, we will briefly summarize the core points of personhood and personal identity that have been discussed up to this point.

As an interim result from the discussion of the constitutive features of personhood, it can be drawn the conclusion that a person is regarded as an agent that has certain mental, rather than singularly human features, wherein rationality is seen as the most fundamental feature. The discussion of the different theories of personal identity suggests that a form of psychological continuity, characterized by overlapping chains of psychological connections, is indispensable to account for the persistence of persons through time. Even though it can not account for all the metaphysical difficulties, in the relevant sense of everyday life, personal identity over time is created by links between present and past provided by autobiographical experience memories and other mental states. These links are seen as providing connections between two discrete, well-defined moments of consciousness. It is beyond the scope of this paper to make an attempt to resolve the ongoing debate on which criterion of personal identity is the most plausible. However, as the brief discussion has shown, we are sympathetic to the reductionist psychological approach which is a widely-held and well-defended view.

It becomes evident that in the discussion of personhood and personal identity a gap remains between the synchronic and the diachronic dimension of a person's life. Although psychological theories of personal identity are based on the assumption that it is a person, rather than a mere biological organism without mental states, who's identity over time is in question, it remains largely unclear how the transition between these timescales—that is, being a person at a discrete point in time, and persisting as a person through time—is mediated, both conceptually and empirically. In order to shed light on this temporal transition, we hereafter focus on habits and decision-making, and argue that therein a conceptually and empirically plausible bridge between personhood and personal identity is to be found.

## Habits and decision-making: a neurophilosophical hypothesis

What are habits? In philosophy of action, habits have been defined as a “pattern of a particular kind of behavior which is regularly performed in characteristic circumstances, and has become automatic for that agent due to this repetition” (Pollard, 2006, p. 57). Standard definitions in psychology are compatible with the philosophical view in the sense that they regard “automaticity and conditioning of repeated acts in stable contexts” (Wood et al., 2002, p. 1282) to be at the core of what habits are. An important feature that distinguishes habits from compulsive behavior is that, in the case of habitual behavior, the person has control over whether or not to perform the habitual action. Based on this conception, habits can explain a vast amount of actions; even more than we would usually assume. This becomes obvious when we think about how much of our lives we spend exercising habits rather than subjecting our actions to deliberation. Starting each day with specific routines, for example getting dressed, brushing teeth, making coffee and so forth. What characterizes habitual behavior is its repetitiveness and automaticity. However, unlike reflexes—for which the same general characteristics apply—habits involve a previous and as the case may be more or less conscious and voluntary acquisition. That is to say, a habit is not something that just passively happens to a person; but rather it is a particular pattern of actions that once has been actively initiated by the person. In light of this, habits can conceptually be seen as a form of actions rather than mere movements. Needless to say, that the level of activeness in the acquisition of different habitual behaviors varies greatly.

Taken together, the criteria for habits extracted from the standard philosophical and psychological definitions are listed in Table 3.

**Table 3:** **Criteria of Habits**.

Component of Conscious Acquisition
Repetition
Automaticity
Conditioning
Stable Contexts
Control

To illustrate the different criteria of habitual behavior, let us consider an example of how habits develop accordingly to the above definition. In the case of running, both if performed professionally as well as in leisure sports, there is a conscious component to the acquisition of the habit to run. At some point, most likely consciously and voluntarily, the person decides to engage in running and to make it a habit by doing this repeatedly. By means of this repetition, let's say the runner decides to run three times a week, the very act of running becomes automatized. However, the involvement of building greater muscle tone that comes along with running is not the same as becoming automatic; rather it makes automaticity possible. That is, a runner becomes able to slowly raise the intensity of running according to the growth of muscle strength and thereby increasing his performance capacity. As a consequence, the runner doesn't have to concentrate anymore on the movements of his legs, arms, etc. while running, but can focus on something else. He could even let his mind wander, or think about something that is completely unrelated to running. The automatized act of running induces a form of learning and improvement in the motion sequence of running. This automaticity leads to a form of conditioning. The person feels the reward of doing sports, gets used to this reward, and gets thereby conditioned to stick to this behavior. It has to be noted here that the reward that comes with doing sports regularly is a feature which is, presumably, based on the voluntariness of engaging in this particular habit. It goes without saying that there are involuntary habits that do not involve reward. For example, slaving away in a mine and excavating stones can become automatic and thus arguably considered to be a habit; nonetheless it most likely does not involve reward. The act of running that occurs with increasing regularity in a well-specified and stable context, as for example in the case of using similar running tracks does further in habitualizing the act of running. Stable context are important in order to make it possible that the automatized act of running can be performed smoothly because the runner doesn't have to adjust to new situations. If, for example, a runner is used to running on tracks and, say, due to having no access to a track while on a trip, so he has to run in the forest, the very act of running might become less smooth because the runner has to adjust his movements to the new environment. Finally, the habit of running is subject to the runner's control. Whenever he decides not to engage in running anymore, for example because he caught a cold and wants to give his body some rest, he can simply decide to do so.

Habits involve particular processes and different levels of decision-making. Following the above analysis, we will first consider the criteria for decision-making that have been examined in current neuroscience. Next, we will examine how these criteria relate to habits.

## Internally and externally guided decision-making within habits

In a recent neuroscientific review paper by Takashi Nakao et al., a distinction between “externally and internally guided decision-making” has been established (Nakao et al., 2012). According to the authors, “most experimental studies of decision-making have addressed situations in which one particular more or less-predictable answer is available” (Nakao et al., 2012, p. 1). It is assumed that in these situations there is one particular correct answer which is almost entirely dependent on external circumstances. Consequently, those kinds of decision processes have been called “externally guided decision-making.” Let us consider an example. Imagine being at a crossroad at which the right-hand road leads to Turin and the left-hand one leads to Pisa. If the goal is to go to Turin, then there is only one correct answer to the decision of which road to take; the answer is entirely dependent on external criteria. The person has to take the right road.

In addition to externally guided decision-making, there are situations in which there is not one correct answer that is based on external circumstances according to which the person decides; but rather, the person has to draw almost entirely on internal resources to make a decision. In these kinds of situations, therefore, the answer depends on the person's own, internal preferences and not on external, circumstantial criteria. Consequently, Nakao et al. call this “internally guided decision-making.” Consider again the example of the crossroad. If the goal is to go to the city you prefer (Turin or Pisa), then there is no externally guided right or wrong answer to the decision of which road to take; it is entirely up to the person's subjective preference whether to take the road to Turin or to Pisa.

In sum, the criteria for externally and internally guided decision-making that have been put forward by Nakao et al. are listed in Table 4.

**Table 4:** **Criteria of externally and internally guided decision-making**.

Externally guided decision-making: The person has to decide mostly relying on externally determined factors. The decision has a single correct answer.
Internally guided decision-making: The person has to decide mostly relying on his/her own internal preferences. The decision has neither a correct nor an incorrect answer.

There is empirical evidence in support of the distinction between internally and externally guided decision-making on a neural level. To test this distinction, Nakao et al. conducted a meta-analysis comparing studies on decision-making that rely on external cues (with high or low predictability of the subsequent gain, i.e., externally guided), with those where no external cues were presented (i.e., internally guided). Interestingly, externally guided decision-making studies yielded significantly stronger activity changes in lateral frontal and parietal regions. Whereas internally guided decision-making studies yielded significantly stronger activity changes in the midline regions; including pregenual anterior cingulate cortex, ventromedial prefrontal cortex, dorsomedial prefrontal cortex, posterior cingulate cortex, and precuneus (see also Northoff, 2014a,b). These data support the distinction between internally and externally guided decision-making on a neural level. The evidence shows that in different decision-making processes that can be characterized as externally and internally guided decisions different brain regions are activated. It has to be noted here that the neural processes underling internally and externally guided decision-making are bilaterally interdependent and reciprocally balanced. That is, activation in the midline regions during internally guided decision-making shows a negative correlation with lateral frontal and parietal regions. However, regardless of the form of decision-making, both regions show a proportional activation in each form of decision-making.

Granting the aforementioned distinction in decision-making, we now turn to ask the question to which degree the criteria of habits reflect internally and externally guided decision-making. Seen from this angle, we will again go through the example of running and examine the degree of externally and internally guided decision-making in the criteria of habits. In this regard, we will refer to elements of habits as “more externally” or “more internally” guided decisions. This, in accordance with the empirical data, suggests that the distinction between both levels of decision-making in the case of habits is not a principal difference, but rather a qualitative difference. It is a difference in levels of internally/externally guided decision-making on a continuum of decisions that range from being almost exclusively external (i.e., there is only one correct answer) to decisions that are almost exclusively internal (i.e., there is no right or wrong answer, only subjective preferences). Furthermore, the distinction between internally and externally guided decision-making in habitual behavior seems to be related to the level in which decisions are made more or less consciously or unconsciously. Concerning this matter, it is useful to distinguish between the process and the outcome of a decision in order to see how these levels are related. While the process of an externally guided decision can be rather unconscious, as for example, in how to adjust movements to certain environmental cues, the outcome of this unconscious process, namely the particular adjustments, can later become conscious and thus may become subject to internally guided deliberation. This gives some reason to suggest that externally guided decision-making is more associated with unconscious processing, whereas internally guided decision-making is more associated with conscious deliberation. Again, this has to be seen as a qualitative difference and not as an all-or-nothing matter.

While acquiring the habit of running, the conscious component in making the decision to run is mostly an internally guided decision, since the idea of engaging in running in the first place is subject to the person's preference. This is in line with the aforementioned assertion that the outcome of a decision, in this example the commitment to engage in the habit of running, is both internally guided and it occurs on a conscious level. Although, there is a more externally guided component to the decision to engage in running as well, that is, to engage in running rather than in, for example cycling, may be influenced by social factors such as the fact that your friends run as well, which is why you like the prospect of joining them. When running is performed repeatedly—in our example let's say the runner decides to run three times a week—the previously conscious component in the decision becomes rather unconscious. That is, the novelty of the decision to engage in running is lost over time. It is rather an externally guided unconscious process, a response to the external stimuli involved in running at specific times. The acquisition of the habit of running was initially a more internally guided conscious decision; however, due to its repetition it becomes a more externally guided unconscious component of habitual behavior. To put it differently, the internally guided decision to engage into running according to the person's preference for this particular sport becomes, due to its repetition, a more externally guided component because in the very act of running it are the external criteria (e.g., the weather conditions, the time schedule etc.) that the runner responds to and not the internal component of deciding which sport to get involved in. The same holds for the automatized component in the process of running. Thereby, the runner does not have to concentrate anymore on the movements of his legs, arms etc. while running, but can focus on something else. There is no conscious, preference dependent decision involved in the very movements of running, but rather an automatized response to external stimuli from the environment in which the running takes place. The component of automaticity in habitual behavior is thus a more externally guided decision-making process because it is merely subject to the environmental circumstances in running. For example, the conditions of the running track due to the weather, the equipment and so forth. Conditioning, on the contrary, is more of an internally guided decision-making process in habitual behavior, since it is based on the internal reward which is related to the preference decision that led to the acquisition of the specific habit in the first place. The element of habitual behavior in running in stable contexts seems to have both levels of internally and externally guided decision-making to it, since the decision to stick to stable contexts is based on the conscious acquisition of the habit to run and is thus more internally guided. Surely, internally guided decision-making is also influenced by the context in which it takes place; however a broader notion of context is meant here, i.e. the social, the political context and so forth. Yet, what we are referring to in the realm of externally guided decision-making is a much narrower notion of context, namely the very concrete environmental conditions by which a decision is shaped. This is why the actualization of running that takes place within a stable context is more externally guided since it is a response to the contextual conditions itself and thus not subject to the runner's preference. The control that one has over the habit of running has also both internally and externally guided elements to it. Control is partly internally guided, because whether or not to continue engaging in the habit of running is based on the person's preference judgment; it is basically a subjective choice. However, this internally guided choice can be dependent on, or at least informed by, externally guided conditions; such as the earlier discussed example of deciding not to run anymore because you caught a cold. The decision to stop running in this case is externally guided to the extent that catching a cold determines whether or not you will physically be able to keep up the habit of running. The external component of catching a cold that influences the decision not to run is externally guided to the extent that it is out of the runner's immediate sphere of control. Whether or not he catches a cold is nothing the runner can do much about—apart from wearing the appropriate clothes according to the weather conditions and so forth. However, once the cold is there, it at least externally informs the decision not to run, because doing so would most likely lead to a worsening of the health condition, which in turn would be at odds with any prudential decision of a rational agent that takes his state of health seriously.

Taken together, the different criteria of habits reflect a balance between internally and externally guided decision-making. Habits, therefore, are neither purely internal nor purely external, but rather they reflect a specific balance between both forms of decision-making.

We now turn to ask what this balance of internally and externally guided components in habitual behavior can tell us about the different timescales that are involved therein. On the one hand, habits are actualized, or take place, at discrete points in time. On the other hand, by repeating the specific actions that take place at discrete points in time, habits take place over time.

## Internally and externally guided decision-making balances synchronic and diachronic elements of habits

The actualization of habits manifests in discrete points in time which indicates a synchronic element of habits. The runner runs Tuesday at 7 PM. This decision-making process is more externally guided, since it largely relies on the external components at this particular point in time in which the decision takes place. For example, how are the weather conditions at this day and how do these conditions guide the decision to run, or influence how to prepare, e.g., to wear a rainjacket?

The repetitiveness of habits over time adds a diachronic element to the actualization of habits at discrete points in time. That is to say, by repeating the actualization of habitual behavior at discrete points in time, the habit takes place over time. The runner runs not only at a particular Tuesday at 7 PM, but he runs every Tuesday at 7 PM. This decision-making process is more internally guided, since it largely represents the person's subjective preference over time and thus involves a diachronic timescale. It is, however, not exclusively internally guided to decide to run every Tuesday at 7 PM, since the runner might only be able to run at 7 PM and not at 11 AM because his work schedule does not permit him to do so. On a neural level internally guided decision-making has been associated with the brain's intrinsic activity, as Nakao et al. point out: “Based on rest-stimulus interaction and the overlap between the network for internally guided decision-making with DMN [Default Mode Network], internally guided decision-making seems to be largely based on intrinsic brain activity” (Nakao et al., 2012, p. 12).

According to the previous analysis, we conclude that habits can be considered to reflect not only a balance between internally and externally guided decision-making, but also a balance between diachronic and synchronic timescales that are involved in the relevant decision-making processes. This means that decision-making in habits already implicates a particular balance between diachronic and synchronic aspects, thus linking two different temporal dimensions.

We now turn to ask what implications the above considerations of decision-making and timescales in habits have for the relation between the philosophical concepts of personhood and personal identity.

## Discussion: philosophical implications of the linkage between personhood and personal identity

The argument which we are going to put forward and discuss in what follows, looks in a semi-formalized way like this:

Premise 1: Personhood is characterized in synchronic terms.
In contrast, personal identity is characterized in diachronic terms.
Premise 2: Habits are a form of ongoing, personalized decision-making processes that have both synchronic and diachronic timescales.
Therefore: Habits link the synchronic and diachronic timescale of a person's life and thus bridge the gap between personhood and personal identity.

**Figure 1:**
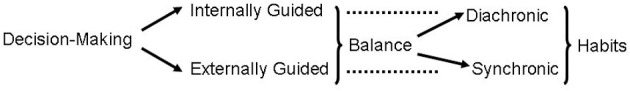
**Habits balance internally and externally guided decision-making and diachronic and synchronic timescales**.

As the foregoing analysis suggests, there is reason to believe that habits are best conceptualized as the sum of personalized, both internally and externally guided decisions that we repeatedly make. This leads us to hypothesize that habits can be seen as the convergence between synchronic and diachronic aspects of a person's life, as illustrated in Figure 1. Personal habits reflect a personalized decision-making process that binds together the synchronic aspects of personhood and the diachronic aspects of personal identity. By so doing, habits, as based on the balance between internally and externally guided decision-making, have the potential to provide an empirically substantiated link between the philosophical concepts of personhood and personal identity. Despite the fact that the actualization of habits takes place synchronically, they nevertheless presuppose, for the possibility of their generation, time in a diachronic sense. Figuratively speaking, the temporal extension of personhood with the recruitment of personal identity is the necessary condition of possibility for the acquisition of habits. More specifically, the acquisition of habits rests both upon a form of rationality, and on psychological continuity, as examined in the accounts of personhood and personal identity. In order to explicate this claim in more detail, we now turn to ask why the acquisition of habits presupposes a form of rationality that has been claimed to be a constitutive condition for personhood, and how this is linked to psychological continuity.

As social psychologists point out, there are self-regulatory benefits of acquiring habits as a way of avoiding the stress, e.g. the time consumption of having to make decisions in similar situations over and over again (Armitage and Conner, 2001). As indicated in the examples given before, persons often rely on habits as an efficient mode of initiating and controlling routines in everyday life. The conscious acquisition of a habit itself, i.e. the conscious decision to keep up a certain pattern of action in stable contexts, therefore, relies on higher-order cognitive functions; namely, on rationality and self-reflectiveness. Once a habit is in place, it is relatively automated; there is no need anymore for a conscious guidance of the habitual behavior. The actualization of a habit is based on a previous, internally guided decision to engage in a particular habit, whereby the concrete performance of this habit becomes automated and is therefore no longer directly subject to self-reflective internally guided decision-making. But rather, the concrete decisions in the situation of performing the habit become responses to external stimuli. The very idea of habit-forming is to avoid the process of deliberative decision-making in recurrent situations for which a rational decision already has been formed. To acquire habits can thus, philosophically speaking, be seen as a form of “practical rationality.” Practical rationality is generally described as the appropriate way of processing information through reasoning; furthermore, it is seen to be the nature of reasons for action and the norms for assessing acts or reasoning leading to action.

To illustrate the argument, consider again a sports example. As a rational agent, you know that it is healthy to do sports regularly. But, unless you purposely form a habit to do sports at specific times, each time doing sports comes to mind, it will bring up the same decision-making process again, and it may thus become difficult to motivate yourself repeatedly. If your goal is to stay healthy, consequently, it is both rational and efficacious to acquire the habit of doing sports. The rational acquisition of habits rests upon a, using Harry Frankfurt's vocabulary, *second-order volition*, i.e., the forming of a will about a will. It rests upon a form of self-reflective deliberation which has been claimed to be constitutive for being a person. Rather than making a rational decision at a specific point in time repetitively, habits are a way to make a rational decision over time and thus link synchronic and diachronic aspects of a person's life.

**Figure 2:**
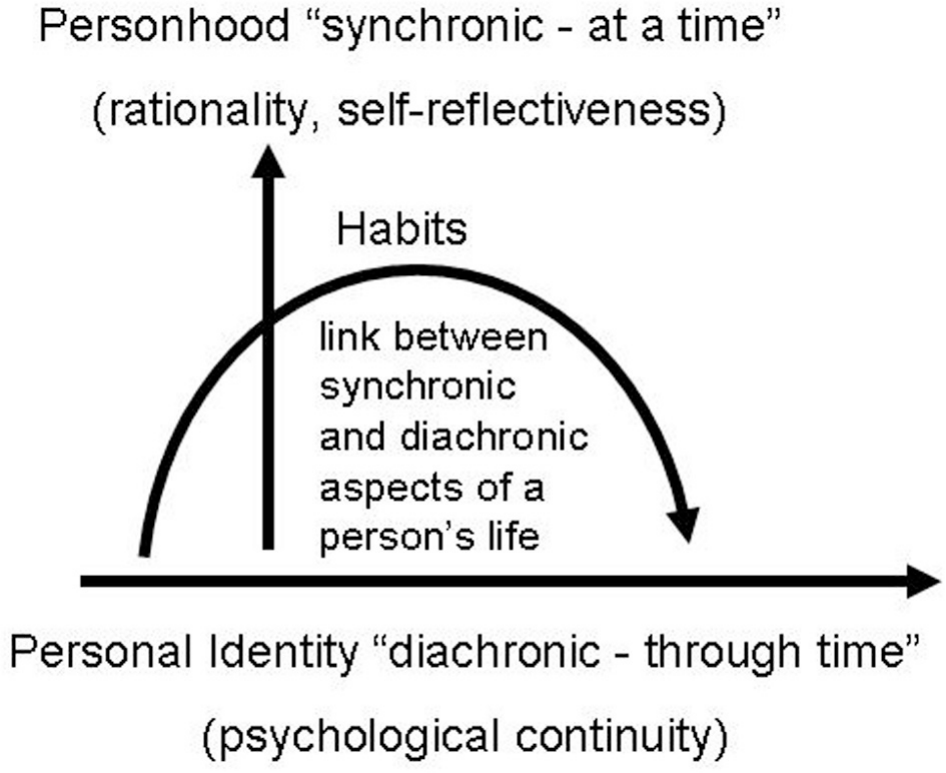
**Habits as a linkage between synchronic and diachronic aspects of a person's life**.

Conceptualizing the repeated intentional actualization of a certain behavior as a habit, however, is only plausible if the person who synchronically performs the particular action persists through time, thus becoming able to repeat the action. How habits bridge the gap between personhood and personal identity is illustrated in Figure 2.

In order to link present habitual behaviors with future ones, that is in order to establish habits, it is necessary that the person at the point of the actualization of the habit is psychological continuous with the person at another point of the actualization of the habit. Putting it more formally: if and only if synchronic person X at time t_1_ is linked through psychological continuity (and is thus identical) with synchronic person Y at time t_2_, an action can possibly become a habit. Seen in this way, acquiring a certain habit becomes a constitutive feature of what it is to be a particular person over time, i.e., what constitutes personal identity.

A person and her identity cannot be narrowly conceived as the synchronic state of psychological features and events alone, but rather a person's identity is inseparable from its familiar modes of behavior, in its familiar environment, which stretches back and forth in time. Habitual actions at a specific point in time emerge from conscious intentions or rather implicit guides that have been developed through past performance, thus linking together the synchronic and diachronic timescales of a person's life. This is true, even more so, if we believe that personal identity depends on the peculiar psychological aspects of a person that manifest in a unique pattern of thoughts and actions which persist through time.

Seeing habits in this light implicates some overlap with what Harry Frankfurt identifies as the constitutive features of being a particular person. Broadly speaking, the notion of distinctively caring about certain lifestyles presupposes the temporal persistence of a particular person. Frankfurt writes: “The outlook of a person who cares about something is inherently prospective; that is, he necessarily considers himself as having a future” (Frankfurt, 1982, p. 260). Habits as deliberatively chosen patterns of behavior are a relevant part of what we care about in our lives and thus account for what it is to be a particular person persisting through time.

In his seminal work on human agency, Michael Bratman makes the case for three core features of agency that can help elucidating our hypothesis that habits bridge the gap between personhood and personal identity. Bratman writes: “We form prior plans and policies that organize our activity over time. And we see ourselves as agents who persist over time and who begin, develop, and then complete temporally extended activities and projects” (Bratman, 2000, p. 35). Accordingly, Bratman claims *reflectiveness*, *planfulness*, and the *conception of our agency as temporally extended* to be the core features of personhood. All of those features are to some relevant degree involved in the acquisition and performance of habits. Pertinent to the linkage between different timescales of a person's life is what Bratman calls “planning agency.” By that he refers to future directed plans of actions that play basic roles in the organization and coordination of our activities over time; the significance of planning for habitual behavior, as discussed in, for example, the scheduling of running, is obvious. Although Bratman does not explicitly discuss habits, he acknowledges that planning typically concerns specific courses of action over time; accordingly he introduces the concept of “policies” as the “commitment [to] a certain kind of action on certain kinds of potentially recurrent occasions” (Bratman, 2000, p. 41). In discussing planfulness and reflectiveness, Bratman draws the attention to the seemingly problematic fact that “one might be reflective about one's motivation at any one time and yet not be a planner who projects her agency over time” (Bratman, 2000, p. 42). Here Bratman's account and our suggestion about habits become importantly connected to psychological continuity relations of personal identity. As mentioned before, psychological continuity does not only hold backwards, but also holds as forward-looking connections to planned habitual actions. That is, habitual behavior can be seen as the link between the forming of a prior intention, for example the plan to run Tuesday at 7 PM and the later execution of this intention. This is only possible if the person who forms an intention is psychological continuous with the person who later executes this intention. Interestingly, sticking with and executing prior plans is not only a passive, or, as it were, automatic psychological fact about persons, but, at the same time, it actively serves to ensure what might be called the “unity of a person over time.” Psychological continuity is thus not only a prerequisite of habitual behavior, but sometimes also an intentional result of a person's activity. In Bratman's words: “[T]he characteristic stability of such intentions and policies normally induces relevant psychological continuities of intention and the like. In these ways our plans and policies play an important role in the constitution and support of continuities and connections characteristic of the identity of the agent over time” (Bratman, 2000, p. 47). Habits, similar to what Bratman calls policies, are thus grounded in their characteristic role of coordinating and organizing a person's identity over time in ways that both constitute and support psychological continuity.

## Concluding remarks

In this paper, we argued on empirically informed grounds that habits bridge the temporal gap between synchronic and diachronic timescales of a person's life, which are exemplified in the philosophical concepts of personhood and personal identity.

In order to substantiate this claim, we first analyzed the seminal concepts of personhood and personal identity in philosophy, thereby carving out the constitutive features of both concepts. According to this analysis, personhood is grounded foremost in rationality, and personal identity is constituted by psychological continuity.

In a next step, we suggested that habits, which are characterized as automatized and conditioned actions that are repeated in stable contexts, can be seen as a specific balance of internally and externally guided decision-making. For this purpose, we drew upon empirical evidence that supports the distinction between internally and externally guided decision-making. On a neuronal level, externally guided decision-making has been associated with lateral frontal and parietal regions. In contrast, internally guided decision-making has been associated with the midline regions. Furthermore, there is reason to believe that externally guided decision-making takes place largely on a synchronic timescale, whereas internally guided decision-making takes place largely on a diachronic timescale.

In a conclusive step, we analyzed how habitual behavior requires and supports both the constitutive features of personhood and personal identity. Based on this analysis, and complemented with what has been established before, namely that habits form a particular balance of internally and externally guided decision-making, we conclude that habits bridge the gap between personhood and personal identity. An empirically informed account of habits can link together what philosophically has so far mostly been described and analyzed separately, and it can therefore open a novel field of philosophical, or rather neurophilosophical investigations.

### Conflict of interest statement

The authors declare that the research was conducted in the absence of any commercial or financial relationships that could be construed as a potential conflict of interest.

## References

[B1] Armitage C. J. Conner M. (2001). Efficacy of the theory of planned behaviour: a meta-analytical review. Br. J. Soc. Psychol. 40, 471–499 10.1348/014466601164939 11795063

[B2] Bratman M. (2000). Reflection, planning, and temporally extended agency. Philos. Rev. 109, 35–61 10.1215/00318108-109-1-35

[B3] Dennett D. (1976). Conditions of Personhood, in The Identities of Persons, ed Rorty A. (Berkeley, CA: University of California Press), 175–196

[B4] Dennett D. (1996). The Intentional Stance. Cambridge, MA: MIT Press

[B5] Frankfurt H. (1971). Freedom of the will and the concept of a Person. J. Philos. 68, 5–20 10.2307/2024717

[B6] Frankfurt H. (1988). The Importance of What We Care About. Cambridge: Cambridge University Press 10.1017/CBO9780511818172

[B7] Frankfurt H. (1982). The importance of what we care about. Synthese 53, 257–272 10.1007/BF00484902

[B8] Kant I. (1785/2012). Groundwork of the Metaphysics of Morals. Cambridge: Cambridge University Press

[B9] Kant I. (1798/2012). Lectures on Anthropology. Cambridge: Cambridge University Press 10.1017/CBO9781139028639

[B10] Korsgaard C. (2009). Self-constitution: Agency, Identity, and Integrity. New York, NY: Oxford University Press 10.1093/acprof:oso/9780199552795.001.0001

[B11] Korsgaard C. (1996). The Sources of Normativity. Cambridge: Cambridge University Press 10.1017/CBO9780511554476

[B12] Lewis D. (1976). Survival and identity, in The Identities of Persons, ed Rorty A. (Berkeley, CA: University of California Press), 17–41

[B13] Locke J. (1694/1975). Of Identity and Diversity, in An Essay Concerning Human Understanding, Chapter XXVII, ed Nidditch P. (Oxford: Clarendon Press), 311–333

[B14] Nagel T. (1971). Brain bisection and the unity of consciousness. Synthèse 22, 396–413 10.1007/BF00413435

[B15] Nagel T. (1986). The View From Nowhere. Oxford: Oxford University Press

[B16] Nakao T. Ohira H. Northoff G. (2012). Distinction between externally vs. internally guided decision-making: operational differences, meta-analytical comparisons and their theoretical implications. Front. Neurosci. 6:31 10.3389/fnins.2012.00031 22403525 PMC3293150

[B17] Noonan H. (2003). Personal Identity. 2nd Edn London: Routledge

[B18] Northoff G. (2001). Personale Identität und Operative Eingriffe in das Gehirn. Paderborn: Mentis

[B19] Northoff G. (2004). Am I my brain? Personal identity and brain identity-a combined philosophical and psychological investigation in brain implants. Philosophia Naturalis 41, 257–282

[B20] Northoff G. (2014a). Unlocking the Brain. Volume I: Coding. New York, NY: Oxford University Press

[B21] Northoff G. (2014b). Unlocking the Brain. Volume II: Consciousness. New York, NY: Oxford University Press

[B22] Nozick R. (1981). Philosophical Explanations. Cambridge: Harvard University Press

[B23] Parfit D. (1971). Personal identity. Philos. Rev. 80, 3–27 10.2307/2184309

[B24] Parfit D. (1984). Reasons and Persons. Oxford: Oxford University Press

[B25] Parfit D. (2007). Is personal identity what matters? in The Ammonius Foundation (South Plainfield, NJ), 1–32 Available online at: http://www.stafforini.com/txt/parfit_-_is_personal_identity_what_matters.pdf

[B26] Perry J. (1972). Can the self divide? J. Philos. 69, 463–488 10.2307/2025324

[B27] Pollard B. (2006). Explaining actions with habits. Am. Philos. Q. 43, 57–68

[B28] Schechtman M. (2010). Personhood and the practical. Theor. Med. Bioethics 31, 271–283 10.1007/s11017-010-9149-6 20607613

[B29] Schechtman M. (2014). Staying Alive–Personal Identity, Practical Concerns, and the Unity of a Life. New York, NY: Oxford University Press 10.1093/acprof:oso/9780199684878.001.0001

[B30] Shoemaker S. (1970). Persons and their pasts. Am. Philos. Q. 7, 269–285

[B31] Shoemaker S. (1984). Personal identity: a materialist's account, in Personal Identity, eds Shoemaker S. Swinburne R. (Oxford: Blackwell), 67–133

[B32] Shoemaker S. (1997). Self and substance. Philos. Perspect. 11, 283–319

[B33] Shoemaker S. (1999). Self, body, and coincidence. Proc. Aristotelian Soc. S73, 287–306 10.1111/1467-8349.00059

[B34] Singer P. (1979). Practical Ethics. Cambridge: Cambridge University Press

[B35] Teichert D. (2000). Personen und Identitäten. Berlin; New York, NY: De Gruyter 10.1515/9783110802320

[B36] Wagner N.-F. (2013). Personenidentität in der Welt der Begegnungen. Berlin; New York, NY: De Gruyter 10.1515/9783110336276

[B37] Wiggins D. (1967). Identity and Spatio-Temporal Continuity. Oxford: Blackwell

[B38] Williams B. (1973). The self and the future, in Problems of the Self, ed Williams B. (Cambridge: Cambridge University Press), 46–64 10.1017/CBO9780511621253.006

[B39] Wittgenstein L. (1921/1961). Tractatus Logico Philosophicus. New York, NY: The Humanities Press

[B40] Wood W. Quinn J. Kashy D. (2002). Habits in everyday life: thought, emotion, and action. J. Pers. Soc. Psychol. 83, 1281–1297 10.1037/0022-3514.83.6.1281 12500811

